# Design of therapeutic education workshops for home haemodialysis in a patient-centered chronic kidney diseases research: a qualitative study

**DOI:** 10.1186/s12882-022-02683-0

**Published:** 2022-02-02

**Authors:** Abdallah Guerraoui, Roula Galland, Flora Belkahla-Delabruyere, Odile Didier, Veronique Berger, Pierre Sauvajon, Christian Serve, Jean Charles Zuriaga, Fréderic Riquier, Agnes Caillette-Beaudoin

**Affiliations:** Department of Nephrology-Dialysis, Calydial, Lyon, France

**Keywords:** Home haemodialysis (HHD), Patient-centered outcomes research (PCOR), Person centred care (PCC), Patient education, Qualitative study

## Abstract

**Rationale & Objective:**

A quarter of patients do not receive any information on the modalities of renal remplacement therapy (RRT) before its initiation. In our facility, we provide therapeutic education workshops for all RRT except for home hemodialysis **(**HHD). The objectives of this study were to identify and describe the needs of CKD patients and caregivers for RRT with HHD and design therapeutic education workshops.

**Setting & participants:**

Two sequential methods of qualitative data collection were conducted. Interviews with patients treated with HHD and doctors specialized in HHD were performed to define the interview guide followed by semi-structured interviews with the help of HHD patients from our center.

**Analytic approach:**

Thematic analysis was conducted and were rooted in the principles of qualitative analysis for social scientists. Data were analyzed by two investigators. Transcribed interviews were entered into RQDA 3.6.1 software for data organization and coding purposes (Version 3.6.1).

**Results:**

In total, five interviews were performed. We identified six themes related to the barriers, facilitators, and potential solutions to home dialysis therapy: (1) HHD allows autonomy and freedom with constraints, (2) safety of the care environment, (3) the caregiver and family environment, (4) patient’s experience and experiential knowledge, (5) self-care experience and impact on life, and (6) factors that impact the choice of treatment with HHD. We designed therapeutic education workshops in a group of patients and caregivers.

**Conclusions:**

Our study confirmed previous results obtained in literature on the major barriers, facilitators, and potential solutions to HHD including the impact of HHD on the caregiver, the experiences of patients already treated with HHD, and the role of nurses and nephrologists in informing and educating patients. A program to develop patient-to-patient peer mentorship allowing patients to discuss their dialysis experience may be relevant.

**Supplementary Information:**

The online version contains supplementary material available at 10.1186/s12882-022-02683-0.

## Background

Early kidney transplantation is the best renal remplacement therapy (RRT) option for many patients with end-stage renal disease (ESRD). However, most patients will need to spend time on dialysis prior to transplantation or when a transplant fails [1–5].

The quality of life and treatment satisfaction are higher with home haemodialysis (HHD). HHD has many advantages:

- Autonomy at home
- Patients Control the flexibility of their dialysis schedule,
- Lowers mortality and morbidity.
- Eliminates transportation to dialysis centers, Reduces travel time/cost
- Improves quality of life, patient mood, sleep, depression
- More independence, personal freedom, time for family and community engagement
- Enhances ability to work
- reduces fatigue
- The Patients who benefit from this system feel much less tired.

Transition among dialysis modalities may be important to maximize quality of life of patients before a kidney transplant, however, home haemodialysis **(**HHD) is rarely chosen. The reasons for changing a patient’s dialysis modality should be assessed considering both short- and long-term benefits and risks as well as the patient’s experience of the transition [6].

The reason for the low use of HHD may be the lack of adapted patient information and education on the benefits it. In one study, a quarter of patients did not receive any information on any modality before the start of RRT including 44% of HHD patients [7]. Moreover, when pre-dialysis information program is developed with patients, a higher proportion of them choose HHD [8]. When shifting to haemodialysis HHD, it is critical to raise patients’ awareness of their condition through appropriate education. This will also increase their acceptance of the need for RRT throughout their life while encouraging self care at the same time [9–13].

Defining a therapeutic education program aimed at addressing benefits, facilitators and barriers of HHD among patients with ESRD adapted to a specific population may thus be relevant to improve the choice of HHD by patients. These factors are differiating and with various priorities depending on representations and culture and it is relevant to conduct local assessment before designing any interventions targeted to a specific population. The objectives of this study were to identify and describe the needs of patients and caregivers of RRT with HHD and to design therapeutic education workshops that could help patients in choosing HHD.

## Methods

### Study design

We conducted a qualitative study with three phases: definition of the interview guide, semi-structured interviews, and design of therapeutic education workshops. The study was conducted with a person-centred research model (PCR) [14–17].

### Definition of the interview guide by two investigators (A.G, and V.B)

The first two interviews were performed with a transplanted patient who had been on HHD treatement and a doctor specialized in HHD treatment. These interviews were used to develop the interview script. The aim of the interview script were to understand the choice of HHD patients, factors influencing their choices and their experiences as well as the obstacles for HHD (see Appendix 1).

### Semi-structured interviews by two investigators (F.B, and O.D)

We conducted semi-structured interviews with HHD patients from our center. Interviews were conducted between February and October 2019 and were analyzed using thematic analysis. In-person interviews occurred in dialysis clinic conference rooms. All participants provided written informed consent to participant in the study. Semi-structured interviews were chosen instead of focus groups to allow deeper data collection and because there was no group dynamics of interest for this study.

Interviews were digitally recorded and professionally transcribed with verbatim. Field notes were taken by the interviewers. Field notes included verbal and non-verbal content that seemed relevant for the author to be recorded. Participant characteristics were self-reported. Patients characteristics that were collected included age, sex, marital status and duration of home dialysis. We conducted semi-structured interviews until we reached data saturation (the point at which little or no new information emerged). The decision was a group decision once no additional subthemes could be identified.

We asked participants to respond to questions about: the factors which triggered their decision to dialyze at home, the obstacles and the difficulties that they thought at that time would hinder the HHD, what are the elements that made them favor being on home dialysis rather than in the dialysis center, do they feel any kind of discomfort or negative reluctances about their current experience, if they had to talk about HHD to another patient, what would they say to them. The interview questions were open and participants were encouraged to provide examples and expand on their responses [18–22].

### Definition of the therapeutic education workshop

The definition of the pedagogical objectives for the therapeutic education workshops was conducted according to the training engineering technique in 4 steps: analyze, design, realize, and evaluate [23].

### Participant selection

Individuals treated with maintenance haemodialysis were eligible if they were at least 18 years old, had been receiving HHD for three months from our center and were French speaking. Hospitalized patients or those who were medically unstable according to their treating nephrologists were excluded. Study staff screened interested individuals for eligibility and obtained written informed consent.

### Data analysis

Semi-structured interviews were transcribed verbatim and verified. Transcripts were entered into RQDA 3.6.1 (2019-07-05) software for data organization and coding purposes (Version 3.6.1) to facilitate data management and analysis (eg, store, review, code, and search data). We used thematic analysis and systematically coded and identified themes inductively from data. To ensure that the range and depth of data were reflected in the analysis, transcripts were independently analyzed by two research team members experienced in qualitative research (AG and PS).

The team identified conceptual patterns among the themes and developed a thematic schema. Concepts were repeatedly discussed by the research team at regular meetings to ensure that the themes reflected the interview data depth. During these discussions, the team returned to the source data (transcripts) to verify findings and ensure that the themes accurately reflected data. Lastly, the research team members along with their patient partners (CS, FR, and JCZ), collaboratively revised the themes until both parties reached an agreement. We reported the study according to the Consolidated Criteria for Reporting Qualitative studies (COREQ) checklist [24].

## Results

In total, 10 HHD patients were included in the study. There was no refusal to participate. Thematic saturation was achieved after five interviews, meaning that no new themes were emerging from the data. Patient interviews were ceased. The mean age was 55.2 years (±14.4). There were four (80%) males and four (80%) were married. The mean HHD duration was 25 months (see Table 1). Two patients started with HHD and three patients were transferred from their haemodialysis in center to HHD.

**Table 1:** Patient characteristics in the study conducted to design therapeutic education workshops for home haemodialysis (HHD) in a patient-centered CKD Research

Patient participants	N (%) or mean ± SD
Patient participants	5
Age (years)	55.2 ± 14.4
Male	4 (80%)
Marital status	
Married	4 (80%)
Not married	1 (20%)
HHD treatement history (month)	28 month (4–68)
Number of HHD session per week	
5	3 (60%)
3	2 (40%)
Interview length (min)	54 ± 12

### Patient interview themes and subthemes

**Fig. 1:**
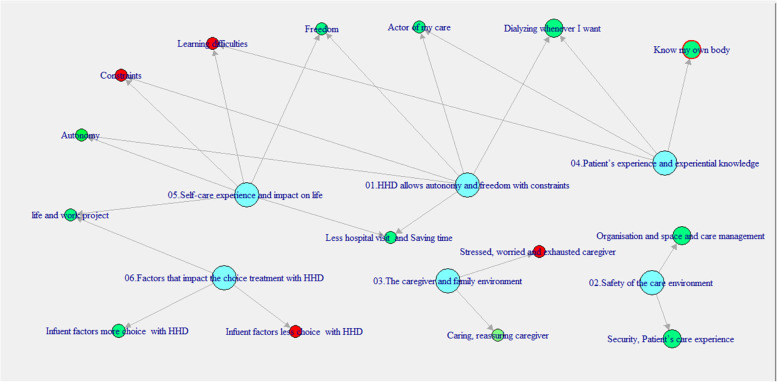
Relationship between the themes and sub-themes of designing therapeutic education workshops for home haemodialysis (HHD) in a patient-centered CKD research. *Themes are in blue, the subthemes positive aspects of HHD are in green and the subthemes negative aspects of HHD are in red

We identified 15 subthemes that were gathered into six major themes. Table 2 displays illustrative quotations for the identified themes and subthemes. The major themes were: (1) HHD allows autonomy and freedom with constraints, (2) safety of the care environment, (3) the caregiver and family environment, (4) patient’s experience and experiential knowledge, (5) self-care t experience and impact on life, (6) and factors that impact the choice of treatment with HHD. Conceptual links among themes and subthemes were illustrated in Fig. 1. The three main themes were 1, 4 and 5 including 9 (60%) sub-themes.

**Table 2:** Themes, subthemes and illustrative quotes in the study conducted to design therapeutic education workshops for home haemodialysis (HHD) in a patient-centered CKD research

Themes	Subthemes	Quotes
1. HHD allows autonomy and freedom with constraints	Active in my care	**Interview 1 Mrs. M [817:865]** I no longer wanted to do dialysis at the center **Interview 2 Mr. B [2225:2337]** I am in charge of my own care. I drive myself to go for dialysis.
	Autonomy	**Interview 1 Mrs. M [3200:3426]** When I am on vacation, I adapt my sessions according to my schedule, I do dialysis early in the morning. Otherwise, when I work, I do dialysis at night. It also depends on my spare time activities such as hiking and biking.
	Doing dialysis whenever I want	**Interview 4 Mr. S [1510:1601]** I can change my dialysis timetable according to my own schedule, and even my sessions. **Interview 5 Mr. Z [3562:3668]** I can connect to it any time I want by continuing to do my dialysis three times a week for 4 h each.
	Less hospital visits and saving time	**Interview 3 Mr. F [601:801]** You don’t think much about the time you waste because of the center’s schedule, waiting for the taxis and traffic congestion. You don’t feel all this right away, but it is very important after all! **Interview 5 Mr. Z [3197:3325]** Staying at home saves me the 70 km round trip between my workplace, the dialysis center and my home. **Interview 5 Mr. Z [4176:4335]** The good thing about home dialysis is that I don’t need to wait for a taxi nor any help to do dialysis. Thus, I can choose the dialysis time, which brings a little sense of freedom for me.
	Freedom	**Interview 4 Mr. S [1601:1614]** I am free to do dialysis whenever I want. **Interview 1 Mrs. M [939:1304]** I went on vacation for 2 weeks with my husband and my daughter. I was able to do dialysis early in the morning and then I had the whole day for us. If I had done dialysis in the center it would have been mandatory in terms of dialysis duration as well as the trip to the center. The nearest dialysis center is located 30 min away from my vacation residence.
	Life and work project	**Interview 1 Mrs M [2048:2379]** The HHD adapts to my lifestyle and not the other way around.
2. Safety of the care environment	Organization, space and care management	**Interview 5 Mr. Z [3740:3846]** Space is needed for the reverse osmosis, the hemodialysis machine as well as the stocked material. We need a whole room dedicated to this. **Interview 1 Mrs. M [4756:5515]** You also need to be well organized regarding the storage. In my dialysis room I have a closet reserved for my dialysis equipment. I always have some compresses at hand, in case of bleeding. I can ask my husband or my daughter, but I prefer to be organized. **Interview 4 Mr. S [2505:2864]** When I get up, I get the hemodialysis machine ready, then I take my breakfast and connect myself. After disconnection, I can eat with my daughter and my wife, no need to wait.
	Security, patient’s care experience	**Interview 3 Mr. F [1502:1700]** When you don’t feel good or have some health issues there is always a way to go back to the dialysis center. There, at least you are surrounded with the staff as well as the other patients. But when you start feeling better go back to home dialysis at once! **Interview 3 Mr. F [3175:3307]** The patient education and training for home dialysis workshops you organized now facilitates my learning.
3. The caregiver and family environment	Stressed, worried and exhausted caregiver	**Interview 2 Mr. B [1797:1899]** Doing dialysis at home weighs on my wife, who is exhausted and stressed. She is also very much worried if my blood pressure is too low. **Interview 2 Mr. B [3607:3787]** I imposed the dialysis on my partner. Doing dialysis at the center has much less impact on her. She is the one who puts up with the illness. Home dialysis means bringing the illness home.
	Caring, reassuring caregiver	**Interview 1 Mrs. M [1827:2000]** My husband agreed to the home dialysis, he is a great help, including with the cycler (disconnects, cleans the cycler, puts the material away and deals with the stock). **Interview 5 Mr. Z [3345:3559]** My partner helps me by setting up my haemodialysis machine when I compress the needle site; she disassembles the haemodialysis machine and cleans it. My partner helps me a lot with my dialysis, a real help. She discontinues my dialysis for me from time to time.
4. Patient’s experience and experiential knowledge	Actor of my care	**Interview 1 Mrs. M [1476:1825]** Doing dialysis during my vacations had no impact or constraint on my family or my activities. This weekend, my husband and I are going to go on a camping car trip to my daughter’s summer camp in the mountain. We put all the material in a closet in our camping car.
	Know my own body	**Interview 3 Mr. F [1196:1449]** The fistula cannulation feels different with the nurse even if she is well trained and is more used to it. I can feel the needle from inside. Even if I miss the cannulation, I feel I am not in, I feel both sides. **Interview 5 Mr. Z [2886:3029]** I think it is very important to have a good knowledge of one’s anatomy and arteriovenous fistula. You feel immediately if you are inside the arteriovenous fistula or beside.
	Dialyzing whenever I want	**Interview 5 Mr. Z [3562:3668]** I can connect any time I want by continuing to dialysis 3 times a week for 4 h each.
	Learning difficulties	**Interview 2 Mr. B [513:583]** It was hard for me to cannulate by myself.
5. Self-care experience and impact on life	Autonomy	**Interview 1 Mrs. M [867:938]** With the cycler I can move, I am not bothered any longer by the lines.
	Constraints	**Interview 2 Mr. B [4340:4431]** Knowing that the accompanying person will be involved in the management. Sometimes it can be oppressive. **Interview 3 Mr. F [1111:1160]** I didn’t want to bring the hospital at home.
	Learnng difficulties	**Interview 1 Mrs. M [2510:2637]** I had difficulties during the first self-cannulation, but I knew it was mandatory for home dialysis. **Interview 5 Mr. Z [2611:2885]** The first cannulation was painful for me. The nurse was telling me to push the needle further but since I have very dry skin, it was hard. I was a little apprehensive about my first cannulation but it was necessary to start it. It was painful and up to now it still hurts at the fistulae.
	Freedom	**Interview 2 Mr. B [947:1118]** More freedom, more flexibility. I can adapt my schedule and even my sessions according to my own schedule/timetable. When I do dialysis in Vienne, I have time constraints.
	Life and work project	**Interview 1 Mrs. M [4297:4432]** You must think about your lifestyle before dialysis, if you like to be autonomous, you must go ahead and be motivated to do HHD.
6. Factors that impact the choice treatment with HHD	Influent factors less choice with HHD	**Interview 2 Mr. B [2679:2996]** Home dialysis is challenging for me. I get up at 5 am every day to run my business, and I do dialysis around 8.30 pm, when my wife gets back from work. She works in Lyon and it takes her an hour to arrive. I go to bed at midnight or 1 am since I am exhausted as much as my wife is. **Interview 4 Mr. S [933:1090]** My wife used to tell me that I was crazy to do dialysis at home, and that it’s too difficult.
	Influent factors more choice with HHD	**Interview 3 Mr. F [1701:1943]** I do dialysis daily, 6 times a week: 3 times for 3 h and 3 other times for 2 or 2 h and a half. I feel much less tired when I do dialysis 3 times a week for 4 h. I recover faster and I can eat better. I can also allow myself an aperitif that I usually wouldn’t take. **Interview 4 Mr. S [767:932]** One day, I went online to get information about home dialysis to better understand how it works. I talked to my doctor who seemed to agree with home dialysis. I gathered a lot of information.

**Fig. 2:**
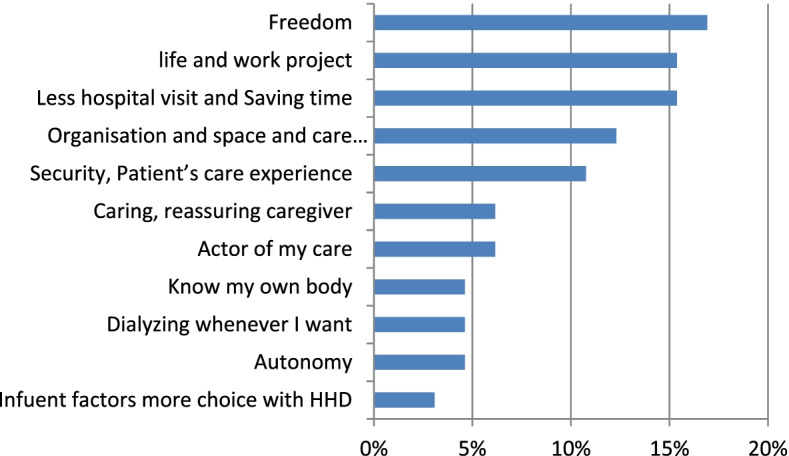
Home haemodialysis **(**HHD) benefits experienced by patients interviewed in a study conducted to design therapeutic education workshops (*N* = 65)

**Fig. 3:**
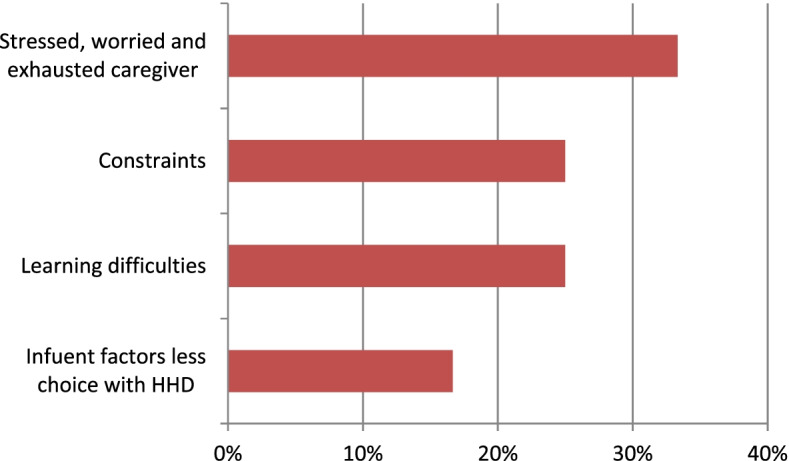
Impact of home haemodialysis (HHD) felt by patients interviewed in a study conducted to design therapeutic education workshops (*N* = 24)

Furthermore, 71% of the subthemes which the patients considered as beneficial and improving quality of life included freedom, life and work projects, less hospital visits and saving time, organisation, space and care management, safety, as well as the patient’s experience of care with 17, 15, 15, 12 and 11% of quotes respectively (Fig. 2). Most subthemes (83%) considered by the patients were negative for the impact on quality of life including being stressed, worried and exhausted as caregivers, constraints and having learning difficulties at 33, 25% and. 25% of quotes respectively (Fig. 3).

### Analyses

Patients described HHD as a technique that increased autonomy and freedom allowing them to be active in their own care, to reduce hospital visits thus saving time, and give flexibility on dialysis schedules.

There was a clear distinction between the expertise of the medical-nurse staff (expert in providing HHD) and the expertise of patients who have been treated with HHD (expert in living with HHD). Patients reported that the medical staff played an important role in improving the theoretical knowledge of HHD treatment. The nursing staff was described as having a leading role in raising awareness and providing support in the choice of HHD technique. However, testimonials and experiences shared by other patients treated by HHD had a major impact in their decision to choose HHD by being more aware of the context of living with HHD treatment.

The skills required for HHD required an apprenticeship for the technique, dialysis machine and the self-puncture of the arteriovenous fistula. The most difficult step in the learning process by all patients interviewed was learning to self-puncture fistula. Furthermore, HHD also requires a new organizational structure at home which may impact family life and particularly impacts the primary caregiver. Involvement of the caregiver from the very beginning of the process seemed critcal for the process to run smoothly.

### Model of therapeutic education workshops

We designed therapeutic education workshops in a group of four patients and four caregivers. An educational assessment of the patient and their caregiver was carried out by a nurse trained in therapeutic education before and after the workshop. The workshop was composed of four educational sequences. Table 3 shows the course of the therapeutic education workshops. Patient’s experience was collected in the form of a video testimony. We applied a pedagogical method (constructivist pedagogy) and pedagogical tools facilitating the expression of the group. A movie on patient experiences is available on our youtube channel. Table 3 regroups the title and the pedagogical objective of each pedagogical sequence.

**Table 3:** Therapeutic education workshop pedagogical sequences for home haemodialysis (HHD) in a patient-centered CKD research

	Title of sequences	Pedagogical objectives
PS1	HHD representations	At the end of the sequence, the patient and their companion will be able to express themselves on their representations and feelings about HHD.
PS2	Benefits and constraints of HHD	At the end of the sequence, the patient and their caregiver will be able to identify the benefits and constraints of HHD.
PS3	Recognize the different types of machines for HHD (conventional, daily generator and cyclers)	At the end of the sequence, the patient and their caregiver will be able to recognize the different types of HHD machines (conventional and cyclers) as well as the one that would be the most adapted to the patient’s needs and expectations.
PS4	Benefits (for me) of the HHD	At the end of the sequence, each participant will be able to recognize the advantages of home hemodialysis to them.

## Discussion

We identified six themes related to the barriers, facilitators, and potential solutions to home dialysis therapy. This includes: (1) HHD allows autonomy and freedom with constraints, (2) safety of the care environment, (3) the impact of the caregiver and family environment, (4) the patient’s experience and experiential knowledge, (5) self-care experience, and impact on life, and (6) factors that impact the choice of treatment with HHD. These themes can be seen with a positive or a negative outlook on performing HHD. Our approach was a person-centered model of care which allowed individualized information. This is why our pedagogical sequence was preceded by an educational assessment. This assessment enabled us to identify the patient’s needs, preferences, therapeutic and life projects. The main strength of our study relied on the qualitive component of it and the two-step design which allowed us to defined content truly adapted to the need of the population. The main limit of the study was the small sample size as well as the representativity of the population which may have been limited.

Our study confirmed previous results in literature obtained about the primary barriers, facilitators, and potential solutions to home dialysis therapy initiation. After receiving education about RRT, patients were more likely to identify the benefits of independent dialysis (autonomy and lifestyle benefits) [25]. Manns et al. conducted a randomized controlled trial in predialysis patients to determine the effect of education on patients’ intention to initiate dialysis in center with self dialysis unit [26]. Patients included in the study were randomized to receive patient-centered education (educational booklets, video, and interactive educational session on self-dialysis) or standard care with education with a multidisciplinary predialysis team. At the end of the study, 82% of the intervention group intended to start independent dialysis versus 50% in the standard care group (*P* = 0.015). Similar results are reflected in another retrospective study which indicated that 55% of patients enrolled in a pre-dialysis education program chose stand-alone dialysis [8].

In another study by Chanouzas, the factors affecting patients choice of dialysis treatment was assessed [27]. The factors considered important by the patients included: the capacity to cope, adaptation of the modality to the lifestyle, distance from the center and the verbal and written information on the modality type. Conversely, the factors that were not considered important by all were: internet use, religious beliefs and the opinions of friends. Patients drew attention to the significance of good information and pre-dialysis education to enable them to choose self-care therapy.

A qualitative study by Seshasai RK [28] identified five themes related to the continuation or discontinuation of HHD. These themes were degree of independence (increased flexibility, burden of therapy), availability of support (emotional and physical support and the burden of a caregiver), technical aspects (familiarity with machine), home environment (ability to organize supplies, space at home), and attitude and expectations (positive or negative outlook about performing HHD) [29].

In 2017, the National Kidney Foundation-Kidney Disease Outcomes Quality Initiative sponsored a home dialysis conference designed to identify barriers to starting and maintaining patients on home dialysis [30]. They identified barriers to the implementation of HHD including patient and caregiver factors such as the lack of adequate education on home dialysis modalities (may not be provided at all to caregivers or patients), psychological, including lack of confidence, fear of self-cannulation, fear of catastrophic events and exhaustion of caregivers [30].

We identified subthemes that can promote the choice of the HHD (freedom, life and work project, less hospital visits and saving time, organisation of space and care management and security, and patient’s care experience). We also identified difficulties encountered with this treatment among the patients treated with HHD. These difficulties were being stressed, having worried and exhausted caregivers, constraints and learning difficulties. Based on the results of our research, we believe that all patients should have information and assistance in choosing replacement therapy including Haemodialysis in center, Self Dialysis Unit, Peritoneal Dialysis, Transplantation and HHD.

There are three relevant topics regarding HHD: the role of the caregiver, the experience of patients already treated with HHD, and the role of nurses and nephrologists in informing and educating. We designed a therapeutic education program that includes four educational sequences that consider our findings. A program to develop patient-to-patient peer coaching that would allow patients to discuss their dialysis experience may be highly relevant. Regarding the patient’s experience, we filmed a partner patient during this research and who is dialysing at home based on the results of the interviews. Although most nephrologists believe that HHD is too complicated and burdensome for most patients with kidney failure [31, 32], this therapeutic education program is now delivered to all patients in our center, as well as other replacement therapies (TX, HD and PD). It is worth noting that a grant was obtained to provide videos in four languages adapted to the population in France (French, English, Spanish and Arabic) which may be used by other centers.

## Supplementary Information

**Additional file 1.**

## Data Availability

The data analysed during this study are included in this published article [Table 1-2-3 and Additional File 1].

## Abbreviations

ESRD: End-Stage renal Disease
RRT: Renal Remplacement Therapy
HHD: Home Heamodialysis
TX: Transplantation
PCC: Person-Centered Care
PCOR: Patient-Centered Outcomes Research
