# Supplementary material for: Design of therapeutic education workshops for home haemodialysis in a patient-centered chronic kidney diseases research: a qualitative study
Source: BMC Nephrol. 2022 Feb 2;23:53. doi: 10.1186/s12882-022-02683-0 (PMC8812054; doi:10.1186/s12882-022-02683-0)
Supplement: Supplementary file 1 — Additional file 1. [file 12882_2022_2683_MOESM1_ESM.docx]

**Appendix 1.** Interview script for the study to design therapeutic education workshops for home haemodialysis (HHD) in a patient-centered CKD Research

Hello/good morning.

I am visiting you on behalf of your nephrologist to get your feedback about home dialysis.

1. Do you agree to share it with us?

[If yes, continue to 2.]

1. Thank you for your time. I would like to know how long you have been on dialysis and how your dialysis is going right now.
2. I would like you to take a few minutes to go back in time to recall the time before you started home dialysis, do you agree?
3. Could you tell me about the factors that triggered your decision to do dialysis at home?
4. What were the obstacles and the difficulties that you thought at that time would hinder the HHD?
5. Now that you have been doing dialysis at home for............what are the elements that made you favor being on home dialysis rather than in the dialysis center?
6. Do you feel any kind of discomfort or negative reluctances about your current experience?
7. If you had to talk about HHD to another patient, what would you say to them?
